# The impact of letrozole administration on oocyte morphology in breast cancer patients undergoing fertility preservation

**DOI:** 10.5935/1518-0557.20200002

**Published:** 2020

**Authors:** Ludmila M.N. Bercaire, Mario Cavagna, Nilka F. Donadio, Andressa R. Rocha, Rafael Portela, Vanessa R. Alves, Thamara B.B. Santos, Felipe Cavagna, Artur Dzik, Luiz H. Gebrim, Eliana A.P. Nahas

**Affiliations:** 1 Human Reproduction Department. Women’s Health Reference Center - Pérola Byington Hospital, São Paulo, SP, Brazil; 2 Gynecology and Obstetrics Department, Botucatu Medical School, UNESP - Universidade Estadual de São Paulo, Botucatu, SP, Brazil

**Keywords:** oocyte morphology, random-start ovarian stimulation, fertility preservation, *in vitro* fertilization

## Abstract

**Objective:**

Patients submitted to oncological fertility preservation with letrozole and gonadotropins seem to present a higher rate of immature oocytes and lower fertilization rates in comparison to infertile patients submitted to IVF cycles with gonadotropins. The aim of this study was to evaluate the influence of letrozole on oocyte morphology in patients with breast cancer submitted to fertility preservation.

**Methods:**

Retrospective analysis performed at a public tertiary hospital in São Paulo, Brazil. The oocytes were retrieved from patients with breast cancer undergoing fertility preservation (n=69), and from infertile women undergoing in vitro fertilization (n=92). We evaluated 750 oocytes obtained from breast cancer patients submitted to ovarian stimulation with letrozole and gonadotropins, and 699 oocytes from patients without breast cancer submitted to ovarian stimulation for *in vitro* fertilization with gonadotropins only due to male factor infertility. The mature oocytes retrieved were analyzed for the presence of refractile bodies, ooplasm color and regularity, central granulation degree, cortical granules, zona pellucida staining and regularity, perivitelline space, presence of vacuoles or abnormal smooth-surfaced endoplasmic reticle and oocyte retraction.

**Results:**

There was a higher incidence of alterations in oocyte morphology in the letrozole group when compared to the control group: increased perivitelline space (*p*=0.007), irregular zona pellucida (*p*<0.001), refractile bodies (*p*<0.001), dark ooplasm (*p*<0.001), granular ooplasm (*p*<0.001), irregular ooplasm (*p*<0.001) and dense central granulation (*p*<0.001).

**Conclusion:**

Letrozole is a risk factor for worse oocyte morphology. However, the clinical impact of ovarian stimulation protocol with combined use of gonadotropins and letrozole for fertility preservation remains unclear in this setting. These data underline the importance of establishing the predictive potential of morphological dimorphisms of human oocytes in IVF outcomes.

## INTRODUCTION

Breast cancer is the leading worldwide cause of death by cancer in women, accounting for 30% of malignancies in the United States. Recent advances in screening and diagnostic methods as well as treatment options have led to a survival rate increase up to 91% over five years in developed countries (American Cancer Society, 2017; Akram *et al*., 2017). Among all women diagnosed with breast cancer in the United States in 2017, about 4% were under 40 years of age (American Cancer Society, 2017).

The high incidence and survival rate of breast cancer in women of childbearing age has led to a considerable concern about fertility preservation treatments. The increased life expectancy of breast cancer patients is attributed to adjuvant treatment with chemotherapeutic agents and hormone therapy (Orvieto, 2015). However, the association of adjuvant treatments has a great impact on ovarian function. Although ovarian failure is not always defined after chemotherapy treatment, many young women evolve to subfertility or infertility (Sonmezer & Oktay, 2006). In this context, the importance of fertility programs is a rising priority in order to improve the quality of life of these patients. It is important to emphasize that ovarian stimulation in these patients does not delay the start of chemotherapy treatment (Reddy & Oktay, 2012). Moreover, it does not seem to be a risk factor for cancer recurrence (Azim *et al*., 2008; Cakmak *et al*., 2013). Thus, cryopreservation of oocytes or embryos are currently the first choice techniques to preserve fertility of patients with breast cancer who will undergo chemotherapy, and the protocol with letrozole is usually applied in patients with hormone-dependent tumors (Kasum *et al*., 2015).

Letrozole is an aromatase inhibitor (AI) developed in the 1990's, as an adjuvant in women with positive estrogen-receptor metastatic breast cancer, as it blocks catalytic conversion of androstenedione to estrone, and testosterone to estradiol (Rodriguez-Wallberg & Oktay, 2010; Halászová *et al*., 2017; Rodgers *et al*., 2017). As a selective and reversible AI, it inhibits proliferation of estrogen-dependent breast cancer cells, promoting reduction of tumor recurrence and higher disease-free survival rates when administered as long-term adjuvant therapy (Rodgers *et al*., 2017; ACOG, 2016; Dahhan *et al*., 2017; Taylan & Oktay, 2017).

Letrozole has also been safely and effectively applied as an ovulation inducer agent in assisted reproduction cycles, due to a reduction in serum estradiol levels that causes the negative feedback and stimulates pituitary production of gonadotropins (Oktay *et al*., 2005; Casper & Mitwally, 2006). It is widely known that successful outcomes of *in vitro* fertilization (IVF) are directly influenced by the presence of both nuclear meiotic division and adequate cytoplasmic parameters to ensure optimal conditions for subsequent development (Alpha Scientists in Reproductive Medicine & ESHRE Special Interest Group of Embryology, 2011; Liu *et al*., 2017). Since nuclear maturity alone is not enough to establish oocyte aspects, there are different morphological, cellular and molecular parameters to evaluate oocyte quality (Liu *et al*., 2017; Rienzi *et al*., 2012; Gilchrist *et al*., 2016). Considering morphological evaluation, an ideal mature oocyte should have a spherical structure enclosed by a uniform zona pellucida, with a uniform translucent cytoplasm, free of inclusions and a single sized-appropriate polar body, with adequate zona pellucida thickness and proper perivitelline space (Alpha Scientists in Reproductive Medicine & ESHRE Special Interest Group of Embryology, 2011; Rienzi *et al*., 2012).

The majority of MII oocytes retrieved after controlled ovarian stimulation protocols displays one or more discrepancies in this described morphological criteria, called oocyte dimorphisms (Rienzi *et al*., 2012; Figueira *et al*., 2010; Sousa *et al*., 2016). Previous studies revealed that outcomes from ovarian stimulation are not impaired by a breast cancer diagnosis, showing similar numbers of total oocytes, MII oocytes and total gonadotrophin dose required (Quinn *et al*., 2017). Nevertheless, previous researches comparing oocytes retrieved from breast cancer patients who received co-administration of letrozole and gonadotropins to oocytes retrieved from infertile patients who received a standard protocol with gonadotropins showed a higher rate of immature oocytes and lower rate of fertilization among the letrozole group, which highlights a possible deleterious effect of letrozole on oocyte maturity (Johnson *et al*., 2013). Regarding the lack of evidence on which stimulation protocol should be used in women with breast cancer and the growing demand for fertility preservation, a large prospective trial is being carried out in order to provide the answers about the effects of various stimulation protocols in women with breast cancer (Dahhan *et al*., 2017). Since there are no published studies regarding oocyte quality on patients undergoing ovarian stimulation protocol with letrozole, the aim of this study was to evaluate the influence of letrozole on oocyte morphological parameters in patients with breast cancer submitted to fertility preservation.

## MATERIALS AND METHODS

### Study design

A retrospective analysis was performed at a Women’s Health Reference Center - Hospital Pérola Byington, a public tertiary hospital in São Paulo, Brazil, through chart analysis and medical records. The sample size was calculated by the Power Sample program, based on the study of Johnson *et al*. (2013), assuming type I errors of 5% and sample power of 80%. Considering the element of study as evaluated oocytes, the estimated sample size was at least 84 elements in the study group and 112 elements in the control group. The purpose of this study was to determine if co-administration of letrozole in conventional ovarian stimulation protocol is associated with oocyte morphological parameter changes in the setting of breast cancer patients undergoing fertility preservation.

### Patients

All women with a recent diagnosis of breast cancer and age < 40 years who underwent ovarian stimulation for fertility preservation between 2015 and 2016 were included in this study (n=69), corresponding to 750 oocytes (study elements). The exclusion criteria were: initiation of chemotherapy prior to ovarian stimulation, age ≥ 40 years, cryopreservation of oocytes due to other types of neoplasms and previous surgery and/or pelvic radiotherapy. Patients submitted to controlled ovarian stimulation for *in vitro* fertilization due to infertility related to male factors and not affected by cancer were defined as controls, and the selection of patients was performed through age pairing (n=92), in order to obtain a similar proportion of 699 oocytes (study elements).

### Controlled ovarian stimulation and oocyte retrieval (Figures 1 and 2)

**Figure 1:**
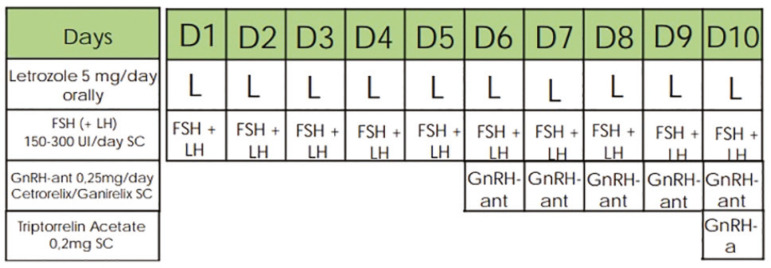
Ovarian stimulation protocol in the letrozole group

**Figure 2:**
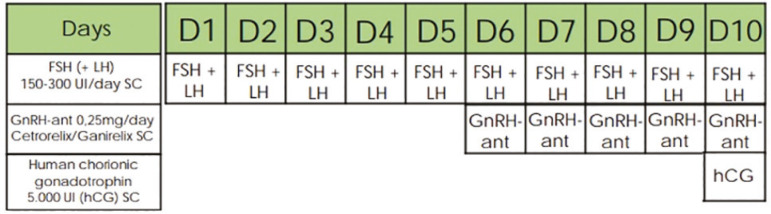
Ovarian stimulation protocol in the control group

Controlled ovarian stimulation for fertility preservation in patients with breast cancer was performed in a random-start protocol, as proposed by our group in a previous study (Cavagna *et al*., 2018), by using letrozole 5 mg/day orally in combination with recombinant or urinary follicle stimulating hormone (FSH) 150-300 IU/day subcutaneously, associated or not with luteinizing hormone (LH), according to ovarian reserve and patient’s age, in order to optimize the ovarian response of these patients. Pituitary blockade was obtained with GnRH antagonist (cetrorelix acetate or ganirelix acetate 0.25 mg/day) subcutaneously in the presence of at least one follicle with ≥ 14 mm diameter. Follicular growth was monitored using transvaginal ultrasound examination until two or more follicles achieved a diameter ≥ 18 mm. GnRH agonist (triptorelin acetate 0.2 mg) was administered subcutaneously to trigger final follicular maturation. Oocytes were collected 36 hours after triggering through transvaginal ultrasound-guided oocyte pick-up.

In the control group, ovarian stimulation was also performed with recombinant or urinary FSH subcutaneously, associated or not with LH in doses varying between 150-300 IU/day, according to ovarian reserve and the patient’s age. Similarly, pituitary suppression was obtained with GnRH antagonist (cetrorelix acetate or ganirelix acetate 0.25 mg/day) subcutaneously in the presence of at least one follicle ≥ 14 mm diameter. Human chorionic gonadotrophin (hCG) 5,000 IU was administered subcutaneously to trigger final follicular maturation, in the presence of two or more follicles with a mean diameter ≥ 18 mm. Oocytes were collected 36 hours after triggering through transvaginal ultrasound-guided oocyte pick-up.

### Oocyte morphology assessment

We kept the retrieved oocytes in culture medium (Irvine Scientific^®^), supplemented with 10% protein and covered with mineral oil (Irvine Scientific^®^) at 37ºC and 6% CO_2_ for 2 hours before removal of the cumulus cells. The surrounding cumulus cells were removed through exposure to a HEPES-buffered medium [N-(2-hydroxyethyl)-piperazine-N'-2-ethanesulfonic acid] containing hyaluronidase (80 IU/mL, Irvine Scientific^®^); and any remaining cumulus cells were mechanically removed by gently pipetting with a hand-drawn Pasteur pipette (Humagen Fertility Diagnostics, VA). We assessed oocyte morphology using an inverted Nikon^®^ microscope under 400X magnification. The oocyte morphology evaluation was performed according to the parameters established in the Istanbul Consensus (Alpha Scientists in Reproductive Medicine & ESHRE Special Interest Group of Embryology, 2011), by qualified and trained embryologists. We assessed the following oocyte abnormalities: cytoplasmic staining and granularity; vacuoles in the ooplasm; refractile bodies in the ooplasm; perivitelline space dimorphisms; cortical granules; zona pellucida (ZP) alterations; endoplasmic reticle aggregations and oocyte shape dimorphisms. Oocytes that had released the first polar body were considered mature and were used for vitrification (letrozole group) or ICSI (control group). We evaluated the morphological parameters of 750 oocytes in the letrozole group and compared to 699 oocytes from the control group.

### Statistical analysis

 We calculated the mean and standard deviation for quantitative variables, frequency and percentage for qualitative variables. We used the Mann-Whitney test for comparison between the groups in relation to quantitative features. We used the Chi-square test to assess the association between the frequencies of categorical features. We ran an analysis to estimate the odds ratio (OR) and 95% confidence interval (CI), considering the group of letrozole to constitute a response compared with the control group as a reference (OR 1.0) to observe the possible associations between letrozole use (dependent variable) and the morphological oocyte parameters (independent variables). The statistical tests were bilateral, and we used a 5% significance level. We used the IBM SPSS (Statistical Package for Social Sciences) program, 23.0 version for the statistical analysis.

### Ethical considerations

 We did not obtain an informed written consent, according to Resolution 446/2012 from the National Health Council. The study was approved by the Research Ethics Committee of the Women’s Health Reference Center - Hospital Pérola Byington (number 888.972, 04/05/2017).

## RESULTS

We evaluated 750 oocytes obtained from 69 breast cancer patients submitted to ovarian stimulation with letrozole and gonadotropins for fertility preservation. In the control group, we analyzed 699 oocytes from 92 patients without breast cancer submitted to ovarian stimulation with only gonadotropins for IVF. The groups were similar regarding mean age (33.1±7.1 years for the control group *versus* 31.5±4.1 years for the letrozole group, *p*=0.210); the total FSH dose (2197.8±875.1IU control group *versus* 2061.5±1207.1 letrozole group, *p*=0.704); total number of oocytes retrieved (10.3±6.1 control group *versus* 12.1±7.9 letrozole group, *p*=0.222); and number of MII oocytes retrieved (7.6±4.8 control group *versus* 9.4±6.6 letrozole group, *p*=0.303) (Table 1).

**Table 1:** Comparison of in vitro fertilization cycle parameters between the letrozole group (n=69 patients) and the control group (n=92 patients)

Variables	Letrozole	Control	*p* value*
Age (years)	31.5±4.1	33.1±7.1	0.210
Total FSH dose (IU)	2061.5±1207.1	2197.8±875.1	0.704
Follicles 14-17 mm (n)	5.8±3.85	5.5±3.2	0.762
Follicles ≥18 mm (n)	5.4±3.4	5.3±3.4	0.946
Retrieved oocytes (n)	12.1±7.9	10.2±6.1	0.222
Retrieved MII oocytes (n)	9.4±6.6	7.6±4.8	0.303

Values are expressed as mean ± SD (Standard deviation).

* Significantly different if *p*<0.05 (Mann-Whitney test)

Regarding oocyte morphological parameters, we found a higher incidence of oocyte dimorphisms in the letrozole group, when compared to the control group: increased perivitelline space (*p*=0.007), irregular zona pellucida (*p*<0.001), refractile bodies (*p*<0.001), dark ooplasm (*p*<0.001), granular ooplasm (*p*<0.001), irregular ooplasm (*p*<0.001) and dense central granulation (*p*<0.001). Other alterations were higher in the control group: cortical granules (*p*<0.001), zona pellucida with altered staining (*p*<0.001) and smooth-surfaced endoplasmic reticulum aggregates (*p*<0.001). There was no statistical difference between the groups in relation to the parameters: retraction (*p*=0.254) and vacuoles (*p*=0.974) (Table 2).

**Table 2:** Comparison of morphological oocyte parameters between the letrozole group (n=750 oocytes) and the control group (n=699 oocytes)

Parameters	Letrozole	Control	*p* value*
Cortical Granules			< 0.001
No	604 (80.5)	292 (41.8)	
Yes	146 (19.5)	407 (58.2)	
Increased Perivitelline Space			0.007
No	490 (65.3)	503 (72.0)	
Yes	260 (34.7)	196 (28.0)	
ZP altered staining			< 0.001
No	743 (99.1)	657 (94.0)	
Yes	7 (0.9)	42 (6.0)	
Irregular ZP			< 0.001
No	641 (85.5)	668 (95.6)	
Yes	109 (14.5)	31 (4.4)	
Refractile Bodies			< 0.001
No	344 (45.9)	385 (55.1)	
Yes	406 (54.1)	314 (44.9)	
Dark Ooplasm			< 0.001
No	522 (69.6)	587 (84.0)	
Yes	228 (30.4)	112 (16.0)	
Granular Ooplasm			< 0.001
No	418 (55.7)	481 (68.8)	
Yes	332 (44.3)	218 (31.2)	
Irregular Ooplasm			< 0.001
No	625 (83.3)	685 (98.0)	
Yes	125 (16.7)	14 (2.0)	
Dense Central Granulation			0.001
No	687 (91.6)	670 (95.6)	
Yes	63 (8.4)	29 (4.1)	
Retraction			0.254
No	696 (92.8)	659 (94.3)	
Yes	54 (7.2)	40 (5.7)	
Vacuoles			0.974
No	717 (95.6)	668 (95.6)	
Yes	33 (4.4)	31 (4.4)	
Smooth ER aggregates			< 0.001
No	749 (99.9)	681 (97.4)	
Yes	1 (0.1)	18 (2.6)	

Values are expressed as number (%).

ZP, Zona Pellucida;

ER, Endoplasmic Reticule.

* Significantly different if *p*<0.05 (Chi-Square test)

Table 3 depicts the association of risk for oocyte dimorphisms in patients submitted to co-administration of letrozole and gonadotropins compared to control patients undergoing conventional stimulation protocol with gonadotropins. The risk of dimorphisms was higher in the letrozole group when compared to the control group (as reference, OR 1.0) in most of the morphological oocytes parameters evaluated (Table 3).

**Table 3:** Association of oocyte dimorphisms risk with letrozole group compared to control group (as reference, OR 1.0)

Parameters	OR	CI 95%	*p* value*
Increased Perivitelline Space	1.4	1.1 - 1.7	0.007
Irregular ZP	3.7	2.4 - 5.5	< 0.001
Refractile Bodies	1.4	1.2 - 1.8	< 0.001
Dark Ooplasm	2.3	1.8 - 2.9	< 0.001
Granular Ooplasm	1.7	1.4 - 2.2	< 0.001
Irregular Ooplasm	9.8	5.6 - 17.2	< 0.001
Dense Central Granulation	2.1	1.3 - 3.3	< 0.001

OR, Odds Ratio;

CI, confidence interval;

ZP, Zona pellucida;

ER, Endoplasmic Reticle.

* Significantly different if *p*<0.05 (Logistic regression)

## DISCUSSION

The major concern associated with conventional controlled ovarian stimulation is the supra-physiological circulating estrogen levels due to the development of large numbers of follicles. Hence, modifications to standard stimulation protocols have been made in an attempt to reduce any detrimental aspect associated with raised serum estradiol levels regarding the risk of cancer recurrence (Rodgers *et al*., 2017; Taylan & Oktay, 2017).

Some previous studies have demonstrated that a letrozole plus gonadotropin protocol is effective for safely inducing patients with breast tumor before initiating adjuvant chemotherapy (Cavagna *et al*., 2018), without increasing rates of its recurrence and without decreasing oocyte yield. Nonetheless, fertility preservation should always be initiated in agreement with the patient’s oncologist (Reddy & Oktay, 2012; Rodriguez-Wallberg & Oktay, 2010; Rodgers *et al*., 2017; Taylan & Oktay, 2017; Ben-Haroush *et al*., 2011; Checa Vizcaino *et al*., 2012; Keskin *et al*., 2014).

During an IVF cycle, daily doses of FSH gonadotropin are used to induce multi-follicular development. Usually, the dose of gonadotrophin used is associated with the number of eggs retrieved; however, the response of individual women is variable (Lensen *et al*., 2018). In the present study, letrozole was used in combination with FSH associated or not with LH in doses varying between 150-300 IU/day according to ovarian reserve and patient’s age. Similar to our findings, Turan *et al*. (2013) showed no difference on number of oocytes retrieved and MII retrieved applying FSH daily injections of 150-300 IU/day. Previous studies displayed a higher number of total oocytes retrieved and total number of MII oocytes with letrozole compared to the standard protocol (Cakmak *et al*., 2013; Dahhan *et al*., 2017). However, other researchers showed lower oocyte maturity rates in breast cancer patients using letrozole compared to IVF patients not affected by cancer (Quinn *et al*., 2017; Johnson *et al*., 2013; Kim *et al*., 2015). We have seen more MII oocytes in the letrozole group (9.4±6.6), than in the control group (7.6±4.8), but this difference was not statistically significant.

In our study, follicular maturation trigger was performed with GnRH agonist in the letrozole group. GnRH agonist trigger for final oocyte maturation is particularly important in cancer patients, in order to decrease the post-trigger estradiol exposure as well as ovarian hyperstimulation syndrome risk compared to an hCG trigger. This syndrome could not only result in a delay in cancer treatment but also amplify the baseline risk for coagulopathy and other cancer-related morbidities (Turan *et al*., 2013; Kim *et al*., 2015; Oktay *et al*., 2010). Besides this, GnRH agonist trigger usually results in a greater mature (MII) oocyte yield without a reduction in clinical pregnancy or live birth rates in cryopreservation cycles (Rodriguez-Wallberg & Oktay, 2010; Rodgers *et al*., 2017; Oktay *et al*., 2010; Pereira *et al*., 2017). The most recent studies showed no differences between these two protocols concerning number of retrieved oocytes (Turan *et al*., 2013; Kim *et al*., 2015). There is no evidence of a difference in live birth rate or ongoing pregnancy rate between frozen-thawed cycles with hCG or GnRH agonist, suggesting that oocyte, embryo quality and morphological characteristics are similar with hCG or GnRH agonist trigger (Orvieto, 2015; Youssef *et al*., 2014). Similarly, to these studies, we found no difference regarding MII oocytes retrieved between GnRH agonist trigger (letrozole group) and HCG trigger (control group).

As far as we know, this is the first study to evaluate the influence of letrozole on oocyte morphology in breast cancer patients. The administration of letrozole is a risk factor for worse oocyte morphological parameters in general, particularly intracytoplasmic effects regarding granular ooplasm.

High quality MII oocytes should have a homogeneous cytoplasm, without inclusions and refractile bodies, with a small perivitelline space comprising a non-fragmented polar body and a colorless and clear zona pellucida (Rienzi *et al*., 2011; 2012). Events such as fertilization, early embryo development and implantation are directly influenced by the normality of nuclear and cytoplasmic oocyte maturation during the preovulatory period (Alpha Scientists in Reproductive Medicine & ESHRE Special Interest Group of Embryology, 2011; Liu *et al*., 2017). Oocyte quality depends on the nuclear and mitochondrial genome, but also on the follicular microenvironment and ovarian conditions, which affect gene transcription and translation processes, and consequently cytoplasmic maturity (Alpha Scientists in Reproductive Medicine & ESHRE Special Interest Group of Embryology, 2011; Rienzi *et al*., 2012; Gilchrist *et al*., 2016). Oocyte dimorphisms are classified into cytoplasmic (granules and/or cytoplasmic inclusions) and extra-cytoplasmic (anomalies in oocyte shape, zona pellucida, perivitelline space and polar body) (Rienzi *et al*., 2012). In our study, oocytes from the letrozole group revealed significantly more dimorphisms than the control group, including some extra-cytoplasmic alterations in zona pellucida and in perivitelline space. In the control group, the zona pellucida showed significantly more altered staining (6% *versus* 0.9%). However, the letrozole group displayed higher frequency of irregular zona pellucida (14.5%) compared to controls (4.4%), but these alterations could be patient-specific, due to the secretion and patterning problems of the glycoprotein matrix (Alpha Scientists in Reproductive Medicine & ESHRE Special Interest Group of Embryology, 2011; Rienzi *et al*., 2012). These morphological aspects should be considered only as phenotypic alterations due to oocyte heterogeneity (Rienzi *et al*., 2011; 2012; Setti *et al*., 2011). Concerning perivitelline space, the letrozole group showed 34.7% of abnormal oocytes, versus 28% in the control group. A large perivitelline space could be related to lower fertilization rate and lower embryo quality, or over-mature oocytes (Rienzi *et al*., 2012). According to the Istanbul Consensus (Alpha Scientists in Reproductive Medicine & ESHRE Special Interest Group of Embryology, 2011), regarding extra-cytoplasmic dimorphisms, inclusions in the perivitelline space is abnormal, but there was no associated evidence to support any specific prognosis (Alpha Scientists in Reproductive Medicine & ESHRE Special Interest Group of Embryology, 2011; Rienzi *et al*., 2012; Figueira *et al*., 2010).

Concerning intracytoplasmic abnormalities, the agreement is that homogeneous cytoplasm is expected, and that non-homogeneous cytoplasm is of unknown biological significance, and based on current evidence, slightly heterogeneous cytoplasm may represent normal variability among retrieved oocytes rather than being a developmental abnormality (Alpha Scientists in Reproductive Medicine & ESHRE Special Interest Group of Embryology, 2011; Rienzi *et al*., 2012).

Small of 5-10 mm vacuoles in diameter, that are fluid-filled but transparent, are unlikely to have a biological consequence, whereas large vacuoles (14 mm) are associated with fertilization failure (Alpha Scientists in Reproductive Medicine & ESHRE Special Interest Group of Embryology, 2011; Figueira *et al*., 2010). Although the presence of granular vacuoles in oocytes was associated with a higher rate of female karyotype abnormalities, the transfer of embryos derived from oocytes with granular vacuoles was associated with higher abortion rates and poor clinical outcomes (Sousa *et al*., 2016). Among intracytoplasmic dimorphisms, smooth endoplasmic reticulum aggregates were considered the most severe abnormality of MII oocytes due the reported outcomes, such as reduced fertilization and clinical pregnancy rates, early fetal demise and imprinting disorders in newborns (Rienzi *et al*., 2011; 2012; Shaw-Jackson *et al*., 2016). These aggregates can be identified as translucent vacuole-like structures in the cytoplasm by phase contrast microscopy (Alpha Scientists in Reproductive Medicine & ESHRE Special Interest Group of Embryology, 2011; Shaw-Jackson *et al*., 2016).

Moreover, some intracytoplasmic dimorphisms as refractile bodies were more frequently seen in the letrozole group than in the control group (54.1% *versus* 44.9%), as well as dark ooplasm (30.4% *versus* 16%), granular ooplasm (44.3% *versus* 31.2%), irregular ooplasm (16.7% *versus* 2%) and dense central granulation (8.4% *versus* 4.1%). Dark zona pellucida was previously associated with lower fertilization, lower implantation and lower pregnancy rates (Shi *et al*., 2014).

While clustering is easily detectable, "granularity" is only seen with optical microscopy modulation, and it is probably that slightly heterogeneous cytoplasm may only represent normal variability (Rienzi *et al*., 2011; Setti *et al*., 2011). Different degrees of cytoplasmic granularity may have diverse outcomes. It seems that oocyte cytoplasmic granularity improves normal fertilization (Rienzi *et al*., 2012; Figueira *et al.,* 2010). However, a dense central granulation is a severe cytoplasmic dimorphism and one should do an oocyte quality evaluation, since it may impair embryo development and implantation potential (Alpha Scientists in Reproductive Medicine & ESHRE Special Interest Group of Embryology, 2011; Ashrafi *et al*., 2015; Setti *et al*., 2011). 

Among intracytoplasmic dimorphisms, smooth endoplasmic reticulum aggregates are the most severe abnormality of MII oocytes (Rienzi *et al*., 2011; 2012; Shaw-Jackson *et al*., 2016). In the control group, there was smooth endoplasmic reticulum in 2.5% of retrieved oocytes, *versus* 0.1% in the letrozole group. Previous studies recommend that one should not use oocytes with this feature for injection (Alpha Scientists in Reproductive Medicine & ESHRE Special Interest Group of Embryology, 2011; Rienzi *et al*., 2011; Shaw-Jackson *et al*., 2016). Nevertheless, studies that are more recent could not find an adverse effect on embryological, clinical or neonatal data for oocytes with smooth endoplasmic reticulum aggregates, since more than 20 children were born from oocytes with this abnormality. Moreover, the exclusion of these oocytes from ICSI cycles causes an increased frequency of transfer cancellation (Ashrafi *et al*., 2015; Shaw-Jackson *et al*., 2014; 2016; Restelli *et al*., 2015).

As mentioned above, evaluation of oocytes’ morphological parameters revealed that the letrozole group had more dimorphisms than the control group. In this study, we did not analyze fertilization or embryo development, as oocytes from breast cancer patients were vitrified, but the probability of a normal fertilization is reduced by the presence of those alterations (Alpha Scientists in Reproductive Medicine & ESHRE Special Interest Group of Embryology, 2011; Setti *et al*., 2011). Based on our results, letrozole is a risk factor for worse oocyte morphology. Nevertheless, some potential biases of this paper must be cited as differences in stimulation protocols, different trigger for final follicular maturation and the breast cancer itself. However, clinical impact of co-administering letrozole and gonadotropins for fertility preservation remains unclear in this setting. These data underline the importance of determining predictive potential of human oocytes morphological parameters and dimorphisms in IVF outcomes.

## CONCLUSIONS

The co-administration of letrozole is a risk factor for worse oocyte morphology, considering higher incidence of dimorphisms as increased perivitelline space, irregular zona pellucida, refractile bodies and dark and granular ooplasm. Although the clinical impact remains unclear in this setting, these findings highlights the importance of observing and determining the predictive potential of human oocyte dimorphisms in IVF outcomes.

**List of abbreviations (in order of appearance):**
1. AI: aromatase inhibitor
2. IVF: in vitro fertilization
3. FSH: follicle stimulating hormone
4. LH: luteinizing hormone
5. GnRH: gonadotropin releasing hormone
6. hCG: human chorionic gonadotrophin
7. OR: odds ratio 8. CI: 95% confidence interval
